# Social prescribing for people living with long-term health conditions: a scoping review

**DOI:** 10.1186/s13643-025-02848-6

**Published:** 2025-05-16

**Authors:** Anna Wilson, Helen Noble, Karen Galway, Julie Doherty

**Affiliations:** School of Nursing and Midwifery, Queen’s University Belfast, Belfast, Ireland

**Keywords:** Social prescribing, Long-term conditions, Health, Wellbeing, Community

## Abstract

**Introduction:**

Social prescribing links people to activities and services typically provided by local voluntary and community sectors to address social determinants of health and wellbeing. People living with long-term health conditions are a target population. This relatively new approach is rapidly expanding, and there is varied evidence regarding how social prescribing is being delivered for people living with long-term conditions. This scoping review aims to report on what is known about the approach for these patient populations.

**Methods:**

Electronic databases MEDLINE, CINAHL, Scopus, Web of Science, and PsycINFO were searched in December 2023, and relevant sources of gray literature in January 2024, with both updated in February 2025. Studies eligible for inclusion included adults (18 +) with long-term conditions engaging with social prescribing in health and community contexts. Studies published in English in any year were included. A data charting template captured key characteristics including reasons for referral, referral pathways, activities and services being utilized, and outcome measures. A descriptive narrative synthesis was conducted, guided by the review questions to explore the current evidence.

**Results:**

Thirty-seven sources of evidence were included. Diabetes was the most common of 65 conditions identified (*n* = 23). The presence of a long-term condition was the most frequent reason for referral (*n* = 30), followed by mental health concerns (*n* = 15), and social isolation or loneliness (*n* = 11). Most referrals were made within primary care (*n* = 33), to a link worker or social prescriber (*n* = 29), who supported participants to access activities and services including exercise (*n* = 22), information, support, and advice (*n* = 19), mental health support (*n* = 15), social and leisure activities (*n* = 15) and condition-specific support (*n* = 14). Wellbeing was the most commonly identified measured outcome (*n* = 23), with studies utilizing the Warwick Edinburgh Mental Wellbeing Scales (*n* = 7) and Wellbeing Star (*n* = 7) most frequently.

**Conclusions:**

While common factors were identified, there is considerable variation in social prescribing approaches for people living with long-term conditions, reflecting the diversity of needs, availability of community services, and necessity for personalized care. Further research is needed to inform the development of evidence-based practice which addresses the complex needs of diverse patient populations and supports access to a broad range of referral pathways.

**Supplementary Information:**

The online version contains supplementary material available at 10.1186/s13643-025-02848-6.

## Introduction

Social prescribing, or community referral, is an approach that links people to a range of activities and support services typically provided by local voluntary and community sectors to address non-medical or social determinants of health and wellbeing [1, 2]. This approach prioritizes people who may require a greater level of social and emotional support to improve mental health and wellbeing than that available in routine care [1]. People are referred by a healthcare professional, often a GP, to a link worker a professional based within a healthcare setting or social prescribing service, who connects individuals to community-based, non-medical support to improve their health and wellbeing [3]. The link worker supports the individual to co-produce a personalized care and support action plan, their own “social prescription,” working towards meaningful goals and enabling access to activities and support within the community [1]. Social prescribing projects such as “ways to wellness” have been developed to specifically target people living with long-term conditions who are most likely to experience health inequalities. This project connects participants with link workers who provide support to achieve personal goals, such as increasing activity levels and improving social connections [4].

Social prescribing is part of the NHS long-term plan [5] and is intended to enable people to take control of their own health and access personalized care when required. It is a key component of the NHS Universal Personalized Care Comprehensive Model [6], which identified a goal to refer at least 900,000 people to social prescribing services by 2023/24; however, evidence indicates that approximately 2.5 million people have been referred in this time period throughout England alone [7]. The implementation of this model is underway across England while the devolved nations of the UK have devised distinct strategies for personalized care, giving people more choice and control over how their care is planned and delivered [8], which in turn have country-specific approaches to social prescribing. NHS England has an additional requirement for primary care networks to provide proactive social prescribing services, which must work with populations experiencing health inequalities [9]. It is widely recognized that social, or non-medical, determinants such as education, housing, social networks, and locality are influential factors in health-related behaviors and outcomes [10] and must be considered alongside clinical interventions when implementing a person-centered approach to care. While UK regions have made strides to integrate the practice of social prescribing within a more holistic approach to care, the approach continues to expand globally [11], with recent reports mapping the adoption of social prescribing policy and practice in 31 countries [12]. The international perspective highlights a need for adaptability of practice across different health systems, identifies the wide range of outcomes being measured throughout social prescribing projects across 13 countries, and calls for further research to develop key common outcomes [13].

Social prescribing aims to support wellbeing by addressing unmet needs through a holistic approach to care, with one of the target populations, people living with long-term health conditions [1]. The most recent Office of National Statistics UK Health Indicators 2019–2020 report states that almost half of the UK population is living with a long-term health condition [14], while one in four of the adult population in England is living with two or more conditions [15]. The impact of this can be detrimental to quality of life and wellbeing [16], and people living with conditions such as cardiovascular disease, diabetes, chronic obstructive pulmonary disease, chronic musculoskeletal disease [17], and kidney disease [18] are at increased risk of depression and anxiety compared to the general population. This in turn has an impact on healthcare services, with the care of people with long-term conditions accounting for an estimated 70% of health and social care spend in England [19]. Evidence suggests that social prescribing has the potential to improve outcomes such as self-esteem, confidence, and mental wellbeing and reduce anxiety and depression [20] by adopting a holistic approach to long-term health conditions [21], with improvements noted in the psychological and social wellbeing of these patient populations [22]. While previous research highlights improvements observed in outcomes, future studies that utilize rigorous methodologies and intervention development processes would be beneficial to evaluate the effectiveness of the approach [23].

As the evidence for social prescribing continues to emerge it is important to consider how the approach is being developed and delivered for specific populations such as those living with long-term health conditions. This scoping review seeks to report on what is known about social prescribing for people living with long-term conditions, identify common factors including reasons for referral, referral pathways into social prescribing services, what outcomes are being measured and how, and identify gaps in the research evidence. A scoping review has been undertaken in an attempt to summarize funding from what is a heterogeneous body of evidence [24] and will enable a broader lens to be taken in regard to identifying and mapping the sources of evidence available. As social prescribing is a relatively novel and emerging approach, consideration will be given to wider forms of literature such as project evaluations which will support the peer-reviewed empirical evidence. Reviewing the evidence concerning social prescribing for people with long-term conditions will also highlight patient populations who are not currently engaging with this approach.

### Objective

The objective of this scoping review is to report on the available evidence in the field of social prescribing approaches for adults living with long-term conditions. The review seeks to:

1. Identify which populations with long-term conditions are engaging in social prescribing schemes, the demographics of participants, and the reason for their referral.
2. Identify and categorize the referral pathways to social prescribing services for people living with long-term conditions.
3. Identify types of activities or services people are being referred to.
4. Identify outcomes being examined, and how are they being measured.

### Methods

This scoping review has been conducted in accordance with the Joanna Briggs Institute methodology for scoping reviews [25]. The Preferred Reporting Items for Systematic Reviews and Meta-Analysis for Scoping Review (PRISMA-ScR) was utilized to develop this scoping review [26]. The PRISMA-ScR checklist contains 20 essential items and two optional items to guide the reporting of scoping reviews [24] (see Additional file 1).

### Protocol and registration

This scoping review was registered at the Research Registry, unique identification number: researchregistry9924, on January 17, 2024.

### Eligibility criteria

Inclusion criteria for the review were identified using the PCC (Population; Concept; Context) framework for scoping review inclusion criteria as recommended by JBI guidelines [25].

### Population

- Participants living with long-term conditions or chronic diseases (over 50% of the study population)
- Over 18 years.

#### Concepts

- Social prescribing—participants are referred to community-based activities or support services to address social determinants, or non-medical factors, that influence health and wellbeing. This approach may also be referred to as community referral, social referral, art prescription, or nature prescription.

### Context

- Health and non-health (community) contexts

In addition to the PCC framework criteria, sources eligible for inclusion included primary quantitative, qualitative, and mixed-method research, journal articles, published reports and guidelines, and sources of gray literature. Only peer-reviewed evidence or evidence sourced from a reputable and credible source have been included such as charitable organizations, healthcare organizations, universities, or governmental organizations. All sources have been published or translated into the English language. Reviews, conference proceedings, editorials, and opinion pieces were excluded from the review, along with sources relating to populations under 18 years old.

### Information sources

To identify potentially relevant documents, the following bibliographic databases were selected due to relevance to the topic area, searched between November and December 2023, and updated in February 2025: MEDLINE, CINAHL, Scopus, Web of Science, and PsycINFO. The electronic database search was supplemented by a review of the reference lists of identified papers to identify other relevant papers. Sources of relevant gray literature were also searched, including the NHS, National Academy of Social Prescribing, Social Prescribing Network, and Social Care Institute for Excellence. Identification of relevant gray literature sources of evidence was undertaken in January 2024 and updated in February 2025.

### Search strategy

A three-step search strategy was utilized, as recommended by JBI [25]. An initial limited search of MEDLINE and CINAHL was undertaken to identify articles on the topic. The text words contained in the titles and abstracts of relevant articles, and the index terms, or subject headings, were used to develop a full-search strategy, which was reviewed by the research team. The final search strategy for CINAHL is presented in Additional file 2. The keywords used within the search strategy were adapted as required for each database and/or information source. The reference list of articles and reports identified for inclusion were manually searched for additional sources of evidence.

### Selection of sources of evidence

Following the search, all identified sources were collated and exported into Covidence systematic review software [27] where duplicates are automatically removed. Studies identified through citation searching were manually uploaded into Covidence. Titles and abstracts were screened by two independent reviewers (AW and JD) for assessment against the inclusion criteria for the review. The full text of selected citations was assessed in detail against the inclusion criteria by four independent reviewers (AW, JD, HN, and KG). Reasons for the exclusion of sources of evidence at full-text stage were recorded and reported. Any disagreements that arose between the reviewers at each stage of the selection process were resolved through consensus and discussion with an additional reviewer if required.

### Data charting process

A tailored data charting template was developed in Covidence to capture relevant information on the key characteristics relating to the scoping review questions and included sections for identification, method, participants, intervention, and outcomes. The template was reviewed and refined by the research team. Data was charted from each source of evidence by AW using a data extraction template.

### Data items

Data from eligible sources of evidence was extracted on identification (e.g., author(s), year of publication, title, project name (if different to title), country of origin and type of source (journal article or gray literature), method (e.g., main aim or objective and study design), participants (e.g., long-term condition, number or percentage of participants with a long-term condition, sex, age, ethnicity, and reason for referral to social prescribing program), intervention (e.g., referral pathway, frequency, and duration of Link Worker contact, activity type) and outcomes (e.g., outcome measures). The data extraction template is available in Additional file 3.

## Synthesis of results

In order to meet the objectives of this scoping review, a descriptive narrative synthesis [28] was conducted, guided by the review questions to identify the current evidence pertaining to social prescribing for people with long-term conditions. Tabular and visual representations of data have been used where appropriate. As the social prescribing practice becomes more prevalent throughout the UK, a range of referral pathways have been utilized to connect participants with voluntary and community support services and activities in their local areas, which are also heterogeneous in nature [29]. Husk [30] has identified a model of four social prescribing pathways; signposting, direct referral, link worker, and holistic, which will be utilized to guide the categorization of pathways identified in this review.

Due to the range of the evidence presented, critical appraisal of individual sources of evidence was not carried out, in accordance with PRISMA-ScR guidance [26].

## Results

### Selection of sources of evidence

**Fig. 1:**
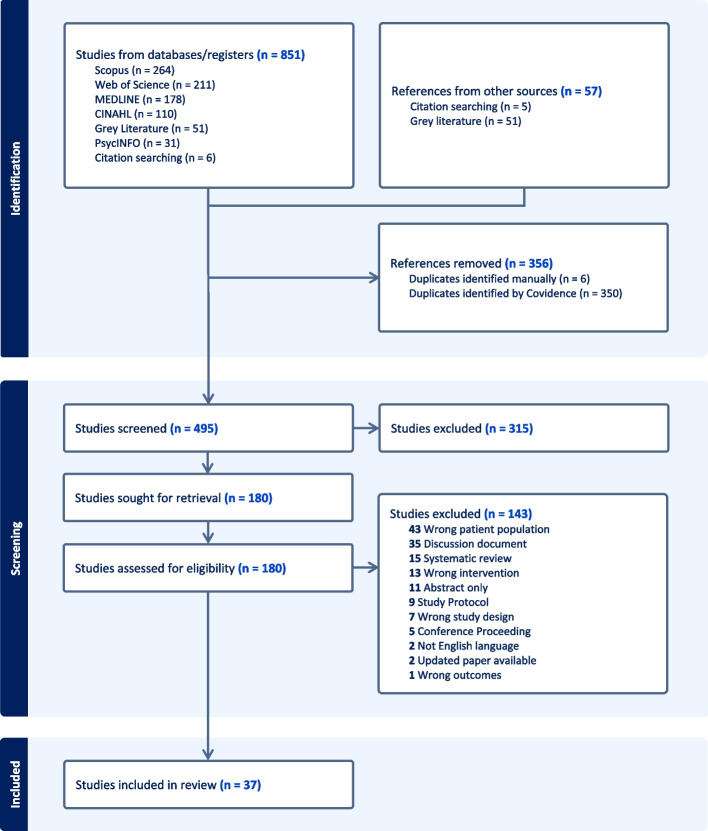
PRISMA flow chart

Of the 908 sources of evidence identified for review, 851 were from database searches, 51 were from gray literature, and 6 sources were from citation searching. Covidence identified and removed 350 duplicates, with a further 6 duplicates manually removed. Titles and abstracts were screened for 495 sources, with 315 deemed irrelevant. The full text of 180 sources of evidence was assessed against the inclusion criteria, and 143 were excluded, primarily due to study populations without long-term conditions or sources that were not primary research. A final total of 37 sources were deemed eligible for inclusion in the scoping review. An overview of the screening process is available in Fig. 1.

### Characteristics of sources of evidence

**Fig. 2:**
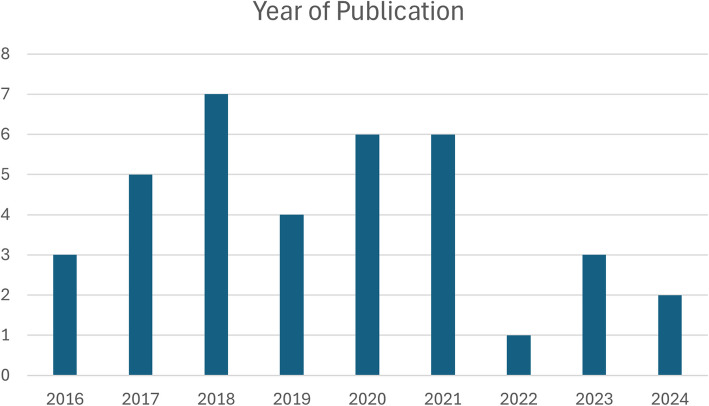
Year of publication

Of the 37 sources of evidence eligible for inclusion, 23 were journal articles and 14 were gray literature. Thirty-one studies were carried out in England including those by case [4] and Moffatt et al. [31], with the remainder carried out in Ireland (*n* = 3) [32–34], Northern Ireland (*n* = 1) [35], Scotland (*n* = 1) [36], and the USA (*n* = 1) [37]. All included studies were published between 2016 and 2024 (Fig. 2).

A wide range of study designs was utilized (Additional file 4: Table S1). The majority were mixed methods (*n* = 18), including evaluations of existing services (*n* = 12), e.g., Bertotti et al. [38], pilot studies (*n* = 2), e.g., Kiely et al. [32], evaluations of pilot studies (*n* = 2), e.g., Mistry et al. [39], and a process evaluation (*n* = 1) [34]. Other designs included pre- and post-study (*n* = 1) [40], action research (*n* = 1) [41], case study (*n* = 1) [42], exploratory case study (*n* = 1) [43], feasibility randomized control trial (*n* = 1) [33], cluster randomized control trial with a parallel mixed-methods process evaluation (*n* = 1) [36], cohort study (*n* = 1) [44], longitudinal cohort study (*n* = 1) [45], and trial within a cohort study (*n* = 1) [46], ethnographic exploration (*n* = 1) [47], multi-methods longitudinal study (*n* = 1) [48], and multi-method evaluation (*n* = 1) [31]. A number of studies utilized qualitative designs including a qualitative study (*n* = 1) [21], a qualitative analysis of a pilot study (*n* = 1) [49], and a qualitative follow-up study (*n* = 1) [50]. The remaining studies utilized quality improvement design (*n* = 1) [35], quantitative evaluation (*n* = 1) [51], and realist evaluation (*n* = 1) [52].

An overview of the included studies and the relevant data is available in Additional file 4: Table S1.

### Synthesis of results

The data retrieved from the 37 included studies was synthesized to meet the four objectives of this scoping review.

### Objective 1: Identify which populations with long-term conditions are engaging in social prescribing schemes, the demographics of participants, and the reason for their referral

The percentage of participants with at least one long-term condition within each study ranged from 52% reported in Ferguson and Hogarth [53] to 100% reported in 29 studies including Kellezi et al. [54]. Four studies identified that the study populations were living with two or more long-term conditions [33, 34, 40, 46]. Of the 37 studies eligible for inclusion, 27 identified specific long-term conditions that were present among the study populations, including Mercer et al. [36] and Mistry et al. [39], with 65 distinct conditions identified. Eight studies did not identify the specific long-term conditions present among their study populations [33–35, 48, 52, 54–56]. The frequency of long-term conditions identified within the studies is presented in Table 1. Of these, diabetes (including types 1 and 2, or both) was the most common long-term condition identified (*n* = 23), e.g., Bird et al. [57] and Moffatt et al. [31].

**Table 1:** Long-term conditions identified within included sources of evidence

Long-term conditions identified	Frequency within studies (*n*)
Angina	2
Anxiety	7
Arterial fibrillation	1
Arthritis	6
Asthma	12
Back pain	1
Back problems	1
Bronchitis	1
Cancer	7
Cardiovascular disease	1
Chronic arterial disease	1
Chronic bronchitis	1
Chronic heart failure	1
Chronic obstructive pulmonary disease	10
Chronic pain	3
Congestive heart failure	2
Coronary heart disease	6
Dementia	7
Dementia—mild impairment stages	1
Dementia (early onset)	1
Depression	9
Diabetes	10
Diabetes (types 1 and 2)	4
Diabetes (type 1)	2
Diabetes (type 2)	7
Eczema/psoriasis	1
Epilepsy	6
Fall risk	1
Fibromyalgia	1
Hard of hearing	1
Heart attack	1
Heart disease	2
Heart failure	5
High blood pressure	5
High cholesterol	3
Hyperlipidemia	1
Hypertension	6
Hypothyroidism	1
Irritable bowel syndrome	2
Kidney disease	5
Learning disability	2
Liver disease	1
Mental health concern	3
Migraine	1
Mini-stroke	1
Motor neurone disease	1
Musculoskeletal problems	1
Neurological condition	1
Obesity	2
Obsessive compulsive disorder	1
Osteoarthritis	1
Osteoporosis	6
Overweight	1
Poor circulation in legs	1
Pre-diabetes	2
Problems with vision	1
Raised cholesterol	1
Respiratory disease	1
Rheumatic disease	1
Rheumatoid arthritis	1
Sciatica	1
Stomach problem/ulcer/etc	1
Stroke	6
Thyroid problem	2

Thirty studies, including Loftus et al. [35] provided sex demographics for the study populations, with the majority (*n* = 24) containing more female participants (between 52%, as reported in Loftus et al. [45], and 90% female in Joseph and Seguin [37]). Seven studies, including Howarth et al. [52], did not specify the sex of the participants.

Participants across the 37 sources of evidence represented a wide range of age groups, with age reported in a variety of formats making it impractical to report an average. However, the data suggests participants tended towards older adults. For example, ten studies, including Palmer et al. [58], reported that most participants were over 70 years. Nineteen studies did not report on the demographic breakdown of participants’ ethnicities, e.g., Simpson et al. [49]. Of the studies that did report on ethnicity (*n* = 16), 15 had a majority of White or White British participants (between 26% reported in the Social Prescribing in Secondary Care project [59] and 100% in Camic et al. [60]) with a single study having a majority of Bangladeshi or Bangladeshi British participants [53].

Regarding the reason for referral, the most common reason reported was the presence of a long-term condition (*n* = 30), including specified diagnoses of dementia (*n* = 4), diabetes (*n* = 2) [38, 42], or cancer [61], with an additional 2 studies using polypharmacy as a proxy for multimorbidity [32, 35]. Other common reasons for referrals were mental health-related concerns (*n* = 15), including low-level mental health concerns or conditions [33, 38], poor mental wellbeing [56], depression [35, 43, 53, 56], anxiety [35, 52, 53, 62, 63], low mood [53, 62, 63], and psychosocial issues [34]. Social isolation [35, 38, 48, 53, 58, 59, 62, 63], loneliness [48, 54], and need for social interaction with others [52, 64] were also identified (*n* = 11). Additional support needs (*n* = 10) were also frequently cited, including advice services for money, debt, and benefits [53, 59, 62, 63], housing advice [53, 59, 64], training and employment [53], help with self-management [46] or significant life changes including recent retirement [49], or stressful issues such as destabilizing events or community issues [59]. Participants in the middle to older age (*n* = 10), as reported in Pollard et al. [47], and being a frequent GP or primary care attender (*n* = 7), e.g., Kiely et al. [33], were also cited as reasons for referral. Referrals to social prescribing schemes and activities to address issues around physical health (*n* = 7) included inactivity [57], engaging in exercise [53], weight management [53, 64], healthy eating [37], and support to address lifestyle risk factors such as smoking and substance addiction and misuse [33, 53, 64]. Two studies did not provide reasons for referral [41, 50].

### Objective 2: Identify and categorize the referral pathways to social prescribing services for people living with long-term conditions

The 37 evidence sources described referral processes in a variety of ways. It was not always specifically clear which discipline of staff had made the referral; however, the following descriptions were used; GPs (*n* = 25), as reported in Kellezi et al. [54] and Mistry et al. [39], other healthcare professionals (*n* = 5), e.g., Palmer et al. [58], practice staff including nurses (*n* = 9), e.g., Polley et al. [64], psychiatrists (*n* = 3), e.g., Ferguson and Hogarth [53], enhanced care teams and programs (*n* = 3), e.g., Howarth et al. [52], community nurses and health workers (*n* = 2) [43, 56], pharmacists (*n* = 2) [53, 56], and psychologists (*n* = 1) [53]. Other means of referral included via charity partners (*n* = 3), e.g., Camic et al. [60], community partners (*n* = 3), e.g., MacMillan Cancer Support [61], secondary care (*n* = 2), e.g., Elston et al. [40], community health and care (*n* = 1) [64], adult social care (*n* = 1) [64], sheltered accommodation partners (*n* = 1) [43], outpatient therapists and occupational therapists (*n* = 1) [49], and through outreach programs (*n* = 1) [61]. Authors reported that participants were also able to self-refer in ten studies including Palmer et al. [58] and Polley et al. [64].

**Fig. 3:**
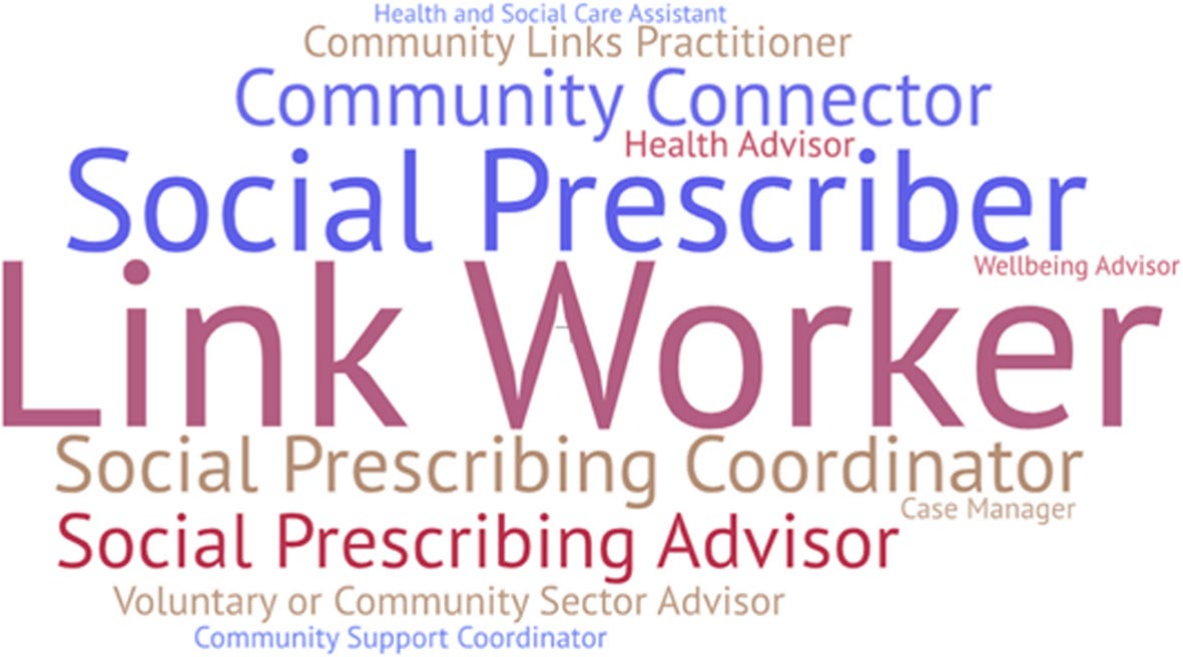
Word cloud of job titles

The majority of the referrals were made to a Link Worker or Social Prescriber (*n* = 29) as reported in Wildman et al. [50] and Case [4]. While Link Worker was the most frequently used job title (*n* = 12), e.g., Kiely et al. [32] and Moffatt et al. [21], this varied considerably, including social prescriber/prescribing coordinator/advisor (*n* = 5), e.g., Palmer et al. [58], community connector (*n* = 3), e.g., Dayson and Leather [63], and Community Health Worker (*n* = 2), e.g., Joseph and Seguin [44]. All job titles included in the review have been presented in Fig. 3, in line with JBI recommendations for the presentation of data [65]. In two studies, referrals were made to an exercise specialist (*n* = 1) [57] or a Community Arts Organization project worker (*n* = 1) [43], and two studies involved initial referral to a health coach, then onwards referral to a Link Worker if required [48, 54]. Additionally, four studies utilized volunteers to support the participants or the Link Workers, holding titles such as dementia care navigators (*n* = 1) [51], wellbeing volunteers (*n* = 1) [52], and volunteer befrienders (*n* = 1) [59].

The social prescribing pathways identified in the included studies have been categorized in line with Husk’s four-pathway model [30], with referrals to activity categorized as follows:

- Signposting
- Direct referral
- Link worker, who connects the participant to the activity
- Holistic, with follow-up and support from the link worker.

Eleven studies, including Loftus [35] and Wakefield [48], utilized a link worker pathway, 25 studies, including Bertotti et al. [38] and Case [4], followed a holistic pathway, eight studies, including Baker and Irving [43], utilized direct referral, and four studies used signposting, including Mercer et al. [36]. Twelve studies, including Howarth et al. [52], utilized a stepped approach, offering multiple pathways depending on an individual’s need. While Husk’s four pathways originate in primary care, alternative referral pathways were also identified, including participants self-referring to the link worker (*n* = 7), e.g., Kellezi et al. [54], self-referring to the activity (*n* = 3), e.g., Chesterman and Bray [41], or referrals that originated in secondary or community care settings (*n* = 6) including Polley et al. [64]. Of the 17 studies that reported on the frequency of contact with the Link Worker, there was a range of contact from a minimum of 6 monthly [31], to no limit [53], depending on the participants’ need. Six studies identified a maximum number of contacts with the Link Worker (*n* = 6), including the social prescribing in secondary care pilot service [59], and five others. Wildman et al. [50] reported the contact as varied. Similarly, of the 17 studies that reported on the duration of Link Worker contact, including Ferguson and Hogarth [53], this ranged widely from 1 month [33, 34] to 4 years [47] depending on the lifespan of the study or project.

### Objective 3: Identify types of activities or services people being referred into

Thirty studies, including Esmene et al. [42] and Munford et al. [45], provided information about the types of activities or services that participants were referred to, which again varied widely. The activities have been categorized into exercise (*n* = 22) including walking groups, e.g., Bertotti et al. [38]; information, support, and advice (*n* = 19) such as help with finances, money, debt, and benefits, e.g., Ferguson and Hogarth [53]; condition-specific support (*n* = 14) including patient support groups, e.g., Moffatt et al. [21]; mental health support (*n* = 15) such as mindfulness, e.g., Palmer et al. [58]; social and leisure activities (*n* = 15) including Men’s Shed as seen in Chesterman and Bray [41]; arts (*n* = 12) including participation in crafting groups, e.g., Dayson and Leather [62]; lifestyle and behavior (*n* = 12) including healthy eating and nutrition advice, e.g., Joseph and Seguin [37]; personal support (*n* = 11) such as accessible taxis, e.g., Bertotti et al. [38]; volunteering (*n* = 10) for charities or community organizations, e.g., Mistry et al. [39]; carer support (*n* = 7) such as befriending, e.g., Palmer et al. [58]; and nature-based activities (*n* = 4) such as gardening, e.g., Moffatt et al. [31]. In 29 studies, participants could be referred to multiple activities or services, and some activities span multiple categories, for example, art therapy for people recovering from stroke [62], creative and cultural befriending for people with dementia [66], or group singing for people with Alzheimer’s [58]. Examples of the activities within each category are provided in Table 2.

**Table 2:** Categories of activities and examples

Activity type (number of studies)	Examples of activity (frequency within studies)
Exercise (*n* = 22)	Walking group (*n* = 7) Dance and movement (*n* = 4) Swimming and water-based exercise (*n* = 3) Yoga (*n* = 3) Gym (*n* = 3) Circuit training (*n* = 2) Netball (*n* = 1) Adapted sports (*n* = 1) Mixed ability sports (*n* = 1) Boxing (*n* = 1) Low-impact exercise (*n* = 1) Tai-chi (*n* = 1) Quiet hours in the gym and swimming pool (*n* = 1) Supported gym sessions (*n* = 1)
Information, support, and advice (*n* = 19)	Finance, money and debt, and benefits advice (*n* = 14) Employment advice and training, e.g., CV writing, return to work support, adult education and training (*n* = 8) Housing advice and support (*n* = 8) Welfare rights (*n* = 5) Learning and skills development (*n* = 5) IT support and training (*n* = 4) Legal advice (*n* = 3) Food referrals (*n* = 2) Management of energy bills (*n* = 1) Safety information and advice (*n* = 1)
Mental health support (*n* = 15)	Psychology, mental health, and wellbeing services (*n* = 10) Mindfulness (*n* = 3) Counseling (*n* = 3) Talking therapies/improving access to psychological therapies (IAPT) (*n* = 2) PTSD support (*n* = 1) Relaxation (*n* = 1) Resilience coaching (*n* = 1) Cognitive behavioral therapy (*n* = 1) Bereavement care (*n* = 1)
Condition-specific support (*n* = 14)	Specialist support or lifestyle programs for long-term conditions (*n* = 4) Patient support and engagement groups (*n* = 3) Dementia support (*n* = 2) Memory cafes (*n* = 2) Stroke support (*n* = 1) Alzheimer’s activity group (*n* = 1) Fibromyalgia support (*n* = 1) Macular degeneration group (*n* = 1) Creative and cultural befriending for people with dementia (*n* = 1) Arts and culture in the community for people with dementia (*n* = 1) Dementia-specific exercise and walking support (*n* = 1) Singing for the brain (*n* = 1)
Social and leisure activities (*n* = 15)	Lunch clubs (*n* = 5) Faith-based attendance and activities (*n* = 3) Men’s Shed (*n* = 3) Libraries and reading group (*n* = 3) Older peoples’ social activities (*n* = 2) Charity activities and support (*n* = 2) Coffee mornings (*n* = 1) Women’s center activities (*n* = 1) Small group activities, e.g., photography, computer club (*n* = 1) Rotary clubs (*n* = 1) Pop-in parlour (*n* = 1) Day centres (*n* = 1)
Lifestyle and behavior (*n* = 13)	Healthy eating and nutrition (*n* = 5) Weight management (*n* = 5) Health trainers (*n* = 2) Lifestyle programs and support (*n* = 2) Manage substance misuse (*n* = 1) Health goal setting (n = 1) Reduce alcohol consumption (*n* = 1) Smoking cessation (*n* = 1)
Arts (*n* = 12)	Arts activities and classes (*n* = 6) Crafts (*n* = 5) Music (*n* = 3) Knitting or crochet group (*n* = 2) Choir or singing group (*n* = 2) Film-making (*n* = 1) Museum object handling (*n* = 1) Creative writing (*n* = 1) Theatre group (*n* = 1) Photography (*n* = 1) Art therapy for people post-stroke (*n* = 1) Arts and culture in the community for people with dementia (*n* = 1) Group singing for people with Alzheimer’s (*n* = 1)
Personal support (*n* = 11)	Support for personal care and independent living, e.g., equipment, adaptations, home aids, cleaning, and decluttering (*n* = 6) Befriending—including condition-specific befriending (*n* = 6) Mobility support—taxi service, accessible, or community transport (*n* = 4) Social services and social care (*n* = 3) Falls prevention (*n* = 3) Escorting and assistance to attend appointments (*n* = 2) Speech and language therapy (*n* = 1) Trusted tradesmen (*n* = 1) Massage therapy (*n* = 1) Walking support (*n* = 1) Disability support (*n* = 1)
Volunteering (*n* = 10)	Volunteering for charity or community organization (*n* = 10) Skills exchange (*n* = 1)
Carer support (*n* = 7)	Support group (*n* = 3) Carer intervention (*n* = 1) Respite (*n* = 1) Care navigation (*n* = 1) Befriending (*n* = 1) Financial support (*n* = 1) Carer wellbeing hub (n = 1)
Nature-based activities (*n* = 4)	Gardening (*n* = 3) Therapeutic horticulture (*n* = 1) Fishing (*n* = 1)

### Objective 4: Identify outcomes examined and how are they being measured

Thirty-one of the included studies identified measurable outcomes of interest, three reported qualitative outcomes [34, 47, 49], and three did not report measurable outcomes [41, 42, 52]. Twenty-four studies utilized multiple outcome measures, with wellbeing as the most frequently measured outcome (*n* = 24). Measures for wellbeing were identified 32 times in these 24 studies, with the Wellbeing star (*n* = 7), as reported in case [4], and short (*n* = 2) and long (*n* = 5) versions of the Warwick Edinburgh Mental Wellbeing Scale, as reported in Elston et al. [40] and the Self-Care Social Prescribing project [66], being the most frequently used outcome measures. Healthcare utilization was another frequently measured outcome (*n* = 12), with the number of contacts or visits to healthcare services identified within 11 studies, including Kellezi et al. [54]. Quality of life was identified as a participant outcome within 12 studies, with the EuroQol 5-Dimension 3 and 5 level (EQ-5D-3/5L) measures most commonly used to measure health-related quality of life, e.g., Moffatt et al. [31]. Health was identified as an outcome in 10 studies, with a range of measures utilized including the EuroQol-Visual Analog Scale (EQ-VAS) (*n* = 3), e.g., Bertotti et al. [38], body mass index (BMI) (*n* = 3), e.g., Polley et al. [64], weight (*n* = 2), e.g., Joseph and Seguin [37], blood pressure (*n* = 2), e.g., Moffatt et al. [31] and glycated hemoglobin level (HbA) (*n* = 2), e.g., Wildman and Wildman [44]. Other outcomes identified throughout the studies were economic measures (*n* = 9), e.g., Panagioti et al. [46], loneliness (*n* = 5), e.g., Wakefield et al. [48], lifestyle (*n* = 4), e.g., Mercer et al. [36], mental health (*n* = 5), e.g., Kiely et al., physical activity (*n* = 2), e.g., Polley et al. [64], social care and support (*n* = 2), e.g., Wakefield et al. [48] and condition-specific measures (*n* = 1), e.g., Panagioti et al. [46] (Table 3).

**Table 3:** Outcome measures identified within included sources of evidence

Outcome (number of studies)	Measure (frequency within studies)
Wellbeing (*n* = 24)	Wellbeing Star (*n* = 7) Warwick Edinburgh Mental Wellbeing Scale (WEMWBS) (*n* = 5) Patient Activation Measure (PAM) (*n* = 4) Measure Yourself Concerns and Wellbeing (MYCAW) (*n* = 3) Distance Travelled Questionnaire (*n* = 3) ICEpop CAPability measure for Adults (ICECAP-A) (*n* = 4) Short Warwick Edinburgh Mental Wellbeing Scale (SWEMWBS) (*n* = 2) Personal/Financial Wellbeing—Office of National Statistics (ONS) (*n* = 2) Subjective Measure of Wellbeing—Canterbury Wellbeing Scale (*n* = 1) HACT Wellbeing Value Calculator (*n* = 1)
Healthcare Utilization (*n* = 12)	No. of contacts with GP/primary care/A&E/hospital/secondary/community care (*n* = 11) Demand for hospital-based health interventions—Hospital Episode Statistics (HES) (*n* = 2) Medication utilisation (*n* = 2) Risk of admission score (*n* = 1)
Quality of life (*n* = 12)	Health-related quality of life—EQ-5D 5/3L (*n* = 12) Quality of life -The World Health Organization Quality of Life Brief Measure (WHOQOL-BREF) (*n* = 1)
Health (*n* = 10)	Global Assessment of Health (EQ-VAS) (*n* = 3) Body mass index (BMI) (*n* = 3) Weight (*n* = 2) Blood pressure (*n* = 2) Glycated hemoglobin level (HbA) (*n* = 2) Cholesterol level (*n* = 1) Rockwood Clinical Frailty Scale (RCFS) (*n* = 1) PROMIS Global Health Short Form v1.2 (*n* = 1) General Measure of Health—Short Form 12 (SF-12) (*n* = 1) Activities of daily living—French Activity Index (*n* = 1) Burden of treatment—Multimorbidity Burden of Treatment Questionnaire (*n* = 1)
Economic (*n* = 9)	Quality-adjusted life years (QALYs) (*n* = 6) Social return on investment (SROI) (*n* = 3) Social capital questionnaire (*n* = 1) Secondary care cost impact (*n* = 1)
Loneliness (*n* = 5)	Loneliness—UCLA Loneliness Scale (ULS-8) (*n* = 2) No. of group memberships (*n* = 2) Campaign to End Loneliness measurement tool (*n* = 1) Social Connectedness—Based on Adult Social Care and Public Health Outcome Framework (ASCOF/PHOF) indicator of social isolation and loneliness (*n* = 1) Community belonging—single item from a population survey of social attitudes (*n* = 1) Community asset participation (*n* = 1) Community belonging—Hayward 1-item (*n* = 1)
Mental health (*n* = 5)	Depression and Anxiety—Hospital Anxiety and Depression Scale (HADS) (*n* = 3) Depression—The Mental Health Inventory (MHI-5) (*n* = 1) Depression—Patient Health Questionnaire (PHQ-9) (*n* = 1) Work and social functioning—Work and Social Adjustment Scale (*n* = 1)
Lifestyle (*n* = 4)	Lifestyle behaviors—smoking, alcohol, substance misuse (*n* = 2) Fruit and vegetable intake questionnaire (*n* = 1) Food literacy questionnaire (*n* = 1) Alcohol Risk—Alcohol Use Disorders Identification Test (AUDIT-C) (*n* = 1)
Physical activity (*n* = 2)	International Physical Activity Questionnaire (IPAQ short) (*n* = 1) Participation in sport—Single Item Sport England Measure (*n* = 1) Physical Activity—General Practice Physical Activity Questionnaire (GPPAQ) (*n* = 1)
Social care and support (*n* = 2)	Social Care Outcomes—Adult Social Care Outcomes Toolkit (ASCOT) (*n* = 1) Social support scale (*n* = 1)
Condition-specific (*n* = 1)	Self-care- The Summary of Diabetes Self-Care Activities (SDSCA) (*n* = 1)

## Discussion

### Summary of evidence

This review explores the current evidence regarding social prescribing for people living with long-term health conditions. Gray literature accounted for over a third of the studies included in this review. This reporting trend in social prescribing research may indicate that this rapidly evolving field relies on more agile and flexible evaluations and reports, rather than the protracted process of publication in traditional academic peer-reviewed journals.

The majority of participants in the studies were older adults, who are more likely to be living with one or more long-term conditions [67]; however, several studies had minimum age requirements which may have resulted in a skewed representation towards older people and may not be fully representative of younger adults with long-term conditions who were not eligible to take part in the included studies. Additional studies involving a broader age range of participants may be necessary to investigate social prescribing across all stages of the life course for people living with long-term conditions. The majority of participants in the studies which reported on ethnicity were from a white ethnic background. There is a need to ensure that principles of equality, diversity, and inclusion are fully integrated into social prescribing practice in order to meet the needs of the individuals engaging with programs and activities [68]. Most studies included more female participants than males. Within the context of social prescribing, it may indicate that males are less likely to seek support for mental health concerns than females [69]. Women are also more likely to experience multiple long-term health conditions than men [70], to engage in help-seeking behavior [71], and are more likely to engage in community activities [72] which may account for this difference in findings. Several studies did identify male-only activities such as Men’s Shed [35, 41, 58] which provides social and community support for men; however, GPs and other healthcare professionals may have missed opportunities to refer more males into social prescribing programs if they did not seek help or indicate that they needed support during routine appointments [73].

A wide range of conditions were identified across the 35 studies included for review, most commonly diabetes, chronic obstructive pulmonary disorder, asthma, dementia, stroke, and a range of cardiovascular issues. Other common long-term conditions impacting adults such as obesity, arthritis, and kidney disease are underrepresented within the findings of this review, indicating that there may be barriers to accessing social prescribing services for these patient populations, and further research to explore the development and implementation of social prescribing for these groups would be beneficial. A number of studies did not specify which particular long-term conditions were present among participants, which limits our understanding of which patient populations have engaged with these services and, as a result, certain conditions may have been underrepresented within this review.

Diabetes is one of the fastest-growing conditions worldwide, with approximately 1 in 11 of the global adult population diagnosed [74]. While diabetes cannot be cured, there are a number of risk factors associated with the condition including obesity, ethnicity, and genetics, and research has shown that type 2 diabetes can be reversed through weight management supported by primary care [75]. For people living with this condition, engaging in structured activities that promote healthier, more active, lifestyles, good diet, and exercise habits through social prescribing schemes may contribute to weight loss and diabetes remission [76]. Included studies that targeted diabetes referred participants to walking groups [38, 42], yoga and netball [38], and healthy eating advice [38]. Exercise interventions were the most common socially prescribed activity across all studies, with walking groups being the most frequently identified, suggesting that this may be an appropriate and accessible activity for a wide range of long-term conditions, as evidence indicates that participating in outdoor walking groups can support physical and psychological health within a social environment [77]. Dementia was also a focus of several included studies [43, 51, 58, 60], with previous research indicating that engaging with the community and voluntary resources, in particular, creative activities including music, arts, and dance programs which promote self-expression and social engagement [78] as being beneficial for the mental health and wellbeing of this patient group [79]. A social prescription may enable more people to access such services and activities. Within this review, participants with dementia were referred to creative activities and interventions including arts and crafts, film-making, dance and movement [43], and museum object handling [60].

While social prescribing programs targeted at people living with diabetes, dementia, and cancer were identified, along with a program driven by patient charity MacMillan Cancer Support [61], other populations with long-term conditions may benefit from a tailored approach to socially prescribed activities which address the nuances of their particular condition. For example, research has highlighted the impact of group singing on respiratory and personal wellbeing for people living with the chronic obstructive pulmonary disorder [80], and arts and mindfulness interventions adapted for people impacted by kidney disease [81, 82] have been recognized as supporting mental wellbeing. Within this review, several studies included activities that were tailored or adapted for participants’ conditions, such as art therapy for people post-stroke [62], and singing for the brain sessions for people living with Alzheimer’s [58]. In order to offer equitable access to activities and services available in the local community, additional consideration may need to be given to the specific requirements of people living with long-term conditions to best meet their needs. This may be reflected in the categories of activities identified within this review, with referrals to condition-specific activities (*n* = 14) and personal support services (*n* = 11) being frequently utilized for participants within the studies.

While most referrals within this review originated in primary care, other referral pathways were identified, including self-referral [60] and those that originated in secondary [59] or community care, such as sheltered accommodation [43] and patient outreach [61], highlighting how integration of social prescribing services is evolving beyond primary care. Social prescribing may have originally been well placed within primary care networks to address patients’ needs; however, those with complex long-term conditions may require alternative referral routes to support their personalized care needs. Enabling healthcare professionals working in specialist secondary care environments to identify individuals who may benefit from a social prescription and make the relevant referral may ensure that the holistic needs of people with long-term health conditions are being met. Expanding the scope of social prescribing programs beyond primary care has been recognized within the literature as having the potential to maximize access for those with non-medical needs [83].

The majority of referrals were made to a Link Worker or similar role, with a wide variety of job titles being utilized. While social prescribing is a novel approach and is still seeking to establish itself within healthcare services, the variation in terms may cause confusion for people who are not familiar with the concept. Future social prescribing approaches may benefit from consistently applied terminology. Husk’s model of social prescribing was used to guide the categorization of pathways [30], with the majority of studies utilizing a Link Worker or holistic approach, which aligns with current NHS England government policy to establish Link Worker-led social prescribing schemes based on primary care networks [5]. Enabling skilled Link Workers to engage with individuals on a more personal level and build a relationship is an important part of the social prescribing process and may positively impact an individual’s engagement with activities and services if they feel regularly supported [50] rather than being signposted or directly referred into activities or services.

The most frequently utilized outcome measure was the Wellbeing Star, designed for use with adults with long-term health conditions, which provides a visual Likert scale to measure an individual’s progress on a “journey of change” [84], and the short and long Warwick Edinburgh Mental Wellbeing scales, which are widely used measures of mental wellbeing for the general population [85]. The brevity, consistency, and validity of these measures indicate that they are appropriate measures for social prescribing programs which aim to support wellbeing, and the visual presentation of the Wellbeing Star may be appealing for participants to track progress. While mental health-related concerns were a frequently cited reason for referral, and around a third of studies referred participants to mental health support services, including mindfulness and counseling, the outcome measures utilized did not reflect this, with just five studies reporting on mental health outcomes [32, 36, 46, 66]. This may be due to the included studies’ focus on addressing general health and wellbeing for populations with long-term conditions and providing a more holistic approach to care, rather than addressing the specific mental health needs of these individuals. Studies which examine social prescribing outcomes for the general population may be more likely to explore mental health outcomes, as highlighted in a recent mapping review of common social prescribing outcomes [13]; however, the evidence recognizes that the prevalence of mental health issues such as depression and anxiety tends to be higher in populations with long-term conditions [17], and further research in this area is required. There was also a focus on healthcare utilization as an outcome of the review. As it is estimated that one in five GP appointments are for non-medical issues such as financial, social, and relationship concerns [86], social prescribing may have the potential to reduce the demand on healthcare services by addressing some of these issues outside of primary care appointments, with a British Medical Association report indicating a 28% reduction in demand for GP services and a 24% fall in A&E attendance for those who had been referred to a social prescribing scheme [87]. Utilizing social prescribing to meet non-medical needs may relieve pressure on national health services; however, populations with long-term conditions may continue to have significant medical needs and require regular support from healthcare services.

While this review has taken a broad scope to the current evidence available within the field of social prescribing for people living with long-term conditions, it is evident that there is a significant need within this population to address social determinants that impact health and wellbeing, with referrals to financial, debt and benefits advice, housing, employment, and personal care support frequently required by study participants. A recent National Institute for Health Research (NIHR) evidence review highlighted that people on the lowest incomes are four times more likely to have multiple long-term health conditions and that socio-economic disadvantage is an underlying driver for health inequality in the UK [88]. From the referrals identified within this review, it is evident that people living with long-term conditions have unmet social, personal, and economic needs and require additional support from health, social, and community care services. The NIHR report [88] identifies addressing the social and economic determinants of health as key to tackling health inequality, which aligns with the aim of a social prescribing approach. Additionally, a social prescribing practice that recognizes the wider factors which contribute to good health reflects the aims of the World Health Organization to address social determinants to advance health equity [89], and works toward the United Nation’s Sustainable Development Goal 3, to ensure healthy lives and promote wellbeing for all at all ages [90]. The social prescribing studies included in this review highlight the wide variety of support required to address factors such as finances, housing, employment, and social inclusion, which play a crucial role in contributing to improving health and wellbeing outcomes for people living with long-term health conditions.

## Limitations

The majority of the sources of evidence included within this review originated in England, with one from outside the UK and Ireland, and no studies identified from non-Western regions. This may be due to several factors, such as the inclusion criteria for studies to be published in the English language, the electronic databases utilized may have been Western-centric, and the sources of gray literature searched were UK-based organizations. The current rollout of social prescribing as an NHS England initiative may account for the skew towards studies conducted in this region, and the terms utilized within the search strategy may not reflect the variation in terminology used globally to describe similar approaches. As social prescribing is a relatively new approach, studies conducted with populations living with long-term conditions may still be in development within current global practice. As this is a scoping review the quality of the studies was not critically appraised to assess and report the risk of bias within the literature, rather the aim was to provide a descriptive overview of how social prescribing approaches are being delivered for people living with long-term health conditions.

## Conclusion

While this review sought to identify common characteristics of social prescribing approaches for people with long-term health conditions, there is a wide range of diversity in approach. Social prescribing must, by its very nature, be diverse, in order to both meet the complex and holistic needs of the people it aims to serve and to reflect the support and services available within the community. There are a variety of populations with long-term conditions currently engaging with services; however, a number of conditions such as cardiovascular disease, kidney disease, multiple sclerosis, and musculoskeletal disorders are underrepresented, and further research into how social prescribing services can be developed and delivered effectively for these groups is recommended. Gaining insight into how social prescribing can support the social and emotional needs of particular patient populations would be beneficial to the development of this approach and will provide crucial evidence to address the social determinants of health impacting these groups. Exploring the expansion of referral pathways could also benefit these populations, who may have limited contact with primary care, and will provide further evidence to support access and engagement with social prescribing as the approach becomes integrated with personalized care planning. Diversity within the evidence base for social prescribing is a recognized issue and is reflected in the heterogenous study designs identified within this review; therefore, it would be advantageous for future studies in this area to develop robust methodologies which add rigor to the current body of evidence. Based on the evidence of this review, it is recommended that future research into the development of social prescribing approaches for people living with long-term conditions recognizes the complex needs of these patient groups, explores how to engage more diverse patient populations, and develops strategies that enable access to a broader range of referral pathways to activities and services which support a holistic approach to personalized care.

## Supplementary Information

Additional File 1. PRISMA-ScR-Fillable-Checklist.

Additional File 2. Search Strategy.

Additional File 3. Data Extraction Template.

Additional File 4. Table S1. Data Extraction Table.

## Data Availability

No new data was generated as part of this review.
