# Supplementary material for: Social prescribing for people living with long-term health conditions: a scoping review
Source: Syst Rev. 2025 May 16;14:114. doi: 10.1186/s13643-025-02848-6 (PMC12085048; doi:10.1186/s13643-025-02848-6)
Supplement: Supplementary file 3 — Additional File 3. Data Extraction Template. [file 13643_2025_2848_MOESM3_ESM.docx]

**Data Extraction Template**

| **Identification** | |
| --- | --- |
| Author |  |
| Year of publication |  |
| Title |  |
| Project Name (if different to title) |  |
| Country of origin |  |
| Type of publication | - Journal Article - Grey Literature |
| **Methods** | |
| Study Design |  |
| **Participants** | |
| Long-term Conditions |  |
| Number or percentage with long-term conditions |  |
| Gender |  |
| Age |  |
| Ethnicity |  |
| Reason(s) for referral |  |
| **Intervention** | |
| Referral pathway |  |
| Frequency of Link Worker contact |  |
| Duration of Link Worker contact |  |
| Activity Type |  |
| Frequency of activity |  |
| Duration of activity |  |
| **Outcomes** |  |
| Outcome measures used |  |
| Timepoints |  |
