# Supplementary material for: Social prescribing for people living with long-term health conditions: a scoping review
Source: Syst Rev. 2025 May 16;14:114. doi: 10.1186/s13643-025-02848-6 (PMC12085048; doi:10.1186/s13643-025-02848-6)
Supplement: Supplementary file 4 — Additional File 4. Table S1. Data Extraction Table. [file 13643_2025_2848_MOESM4_ESM.docx]

**Table 1. Data Extraction Table**

| **Author & Year** | **Title** | **Study Design** | **Long-term Condition(s)  Number (or %) of participants with LTCs** | **Participant Demographics   Sex Age Ethnicity** | **Reasons for Referral** | **Referral Pathway  Frequency and duration of link worker contact  Referral Pathway Model** | **Activity Type** | **Outcome Measures Used** |
| --- | --- | --- | --- | --- | --- | --- | --- | --- |
| Baker and Irving 2016 [43]  England  Journal Article | Co-producing Approaches to the Management of Dementia through Social Prescribing | Exploratory case study | Dementia  63% | Not specified  Not specified  Not specified | Presence of dementia and depression | GPs, community health workers and sheltered accommodation managers referred individuals to Community Arts Organisation project worker to discuss the project and assess patient suitability.  N/A  Direct referral (2) Community | Arts-based interventions (focus on dance, movement, crafts, and filmmaking) | Wellbeing Distance Travelled questionnaire |
| Bertotti et al. 2020 [38]  England  Grey Literature | An evaluation of Social Prescribing in the London Borough of Redbridge: final evaluation report | Mixed methods evaluation | Diabetes (type 2)  75.5% | Male 36.2% Female 63.8%  18-24 1.9% 25-34 7.5% 35-44 12.3% 45-54 12.3% 55-64 13.2% 65-74 13.2% 75-84 23.6% >=85 16.0%  White British 40.4% White other 6.2% Mixed 2.2% Black or Black British 7.3% Asian or Asian British 34.3  Other 9.6 | Type-2 Diabetes Low level mental health Social Isolation Carers | GPs refer patients to Social Prescribing Coordinator/Advisor for assessment via initial phone call followed by support sessions. Health and Wellbeing Buddies accompany patients with high support needs to access services.  Up to 5 x one-to-one sessions over 12-week period  Holistic (3+) | Social networking - Age UK unspecified referrals, lunch clubs, attendance to mosque Mental well-being/mental health - talking therapies, mindfulness, counselling, PTSD Training - IT support, confidence building Physical activity - yoga, netball, walking groups Health advice - healthy eating, MS, Age UK fall prevention Mobility support - Taxicard Benefit advice - food vouchers and general legal advice Employment advice Other support services Volunteering Carers support Arts and music - library, art classes Housing benefits Social services Disability support Gardening Support for vulnerable migrants | Quality of Life (EQ5D-5L) General Health (EQ VAS)  Short Warwick Edinburgh Mental Wellbeing Scale (SWEMWBS) Campaign to End Loneliness measurement tool  Social Capital questionnaire Social Return on Investment SROI |
| Bird et al. 2019 [57]  England  Journal Article | General practice referral of "at risk" populations to community leisure services: applying the RE-AIM framework to evaluate the impact of a community-based physical activity programme for inactive adults with long-term conditions | Mixed methods evaluation | Diabetes (type 2) Pre-diabetes Hypertension Obesity  100% | Female 63% Male 37%  18-34 2.8% 35-50 7.0% 51-69 38.0% 70+ 51.3%  White 96.3% Mixed ethnic group 0.5% Black British 0.2% Asian 0.3% Asian British 0.3% Other 0.7% | Presence of LTC Inactivity | GP or healthcare professionals make referral to exercise specialist within physical activity in local leisure centres and community-based venues. Further signposting to local physical activity classes after completion of programme.  N/A  Signposting (1) Direct referral (2) | Physical activity - circuit training, walking groups, adapted sports | Weekly physical activity - International Physical Activity Questionnaire (IPAQ short) Participation in sport - Single Item Sport England Measure Warwick Edinburgh Mental Wellbeing Scale (WEMWBS) BMI |
| Camic et al. 2022 [60]  England  Journal Article | Subjective wellbeing in people living with dementia: exploring processes of multiple object handling sessions in a museum setting | Mixed methods design | Dementia - mild impairment stages  100% | Not specified  50 years +  White 100% | Dementia diagnosis | Patients self-refer through recruitment via local dementia settings and charities, online, day centres, waiting rooms, dementia involvement group and dementia research database  N/A  Self-referral to activity | Museum object handling | Subjective Measure of Wellbeing - Canterbury Wellbeing Scales |
| Case 2021 [4]  England  Grey Literature | Ways to Wellness The First Six Years: Approach, Findings and Learning | Mixed methods evaluation | Chronic obstructive pulmonary disease  Asthma Diabetes (type 1 or 2) Heart disease (coronary heart disease or congestive heart failure) Epilepsy Osteoporosis  100% | Not specified  40-74 years  Not specified | Age (40-74) Living in area of high deprivation Presence of 1+ LTC. | GPs and other frontline HCPs refer patients to link workers, who work with clients to identify personally meaningful goals, working towards achieving them through an agreed action plan. Link workers signpost clients to community services and groups when it aligns to their goals, supporting clients to access and engage, if needed.   Not specified  Link Worker (3) Holistic (3) | Healthier behaviours (exercise, healthy eating, weight loss)  Financial or benefits advice  Support for long-term condition management  Housing advice or support  Mental health support  Support for personal care (e.g. equipment, adaptations, support)  Other activities or groups  Addiction services  Volunteering or employment services  Transportation services | Wellbeing Star Secondary care cost impact No. of GP visits |
| Chesterman and Bray 2018 [41]  England  Journal Article | Report on some action research in the implementation of social prescription in Crawley. Paths to greater wellbeing: "sometimes you have to be in it to get it" | Action research | Depression Anxiety Obsessive Compulsive Disorder Dementia (early onset) Diabetes  100% | Not specified  18+   Not specified | Not specified | GP/psychiatrist/self-referral to wellbeing activities  Not specified  Direct referral (2) Self-referral to activity | Craft activity Coffee mornings Recovery classes Volunteering Dementia support group Sports Men's Sheds Mental health services Creative writing | N/A |
| Dayson and Bennett 2016 [56]  England  Grey Literature | Evaluation of Doncaster Social Prescribing Service: understanding outcomes and impact | Mixed-methods evaluation | Not specified  640 (60%) | Female 634 (59.9%) Male 420 (39.7%) Unknown 4 (0.4%)  <30 - 101 31-50 - 199 51-60 - 159 61-70 - 128 71-80 - 163 81-90 - 205 >90 - 56  Not specified | Address effects of LTC or mental health condition Mid to moderate depression Poor mental wellbeing Frequent GP attender | SP Service's Advisors receive client referrals from GPs, community nurses and pharmacists, and provide them with support to access a range of voluntary, community and statutory services to meet any additional needs that are identified. Advisors meet with the client in their own home to discuss referral options, attend initial visits, and volunteers support subsequent visits and support clients to start accessing other services and groups.  Not specified  Holistic (3+) Community | Volunteering Debt and housing advice Music and dance workshops | Health Related Quality of Life - EQ-5D (3L) Social Connectedness - Based on Adult Social Care and Public Health Outcome Framework (ASCOF/PHOF) indicator of social isolation and loneliness Financial Wellbeing - ONS Cost-effectiveness - Quality Adjusted Life Years (QALYs) |
| Dayson and Leather 2018 [63]  England  Grey Literature | Evaluation of Hale Community Connectors Social Prescribing Service 2017 | Mixed methods evaluation | Depression Anxiety Arthritis  Diabetes Asthma Chronic pain  77% | Female 69% Male 31%  Under 25 6% 25-44 32% 45-65 35% 65-84 20% 85+ 6%  White British 47% Pakistani 40% Other ethnic origin 13% | Anxiety and low mood Social isolation Presence of LTC Frequent GP attender Money and debt | Patient referred from GP to Community Connector who identifies patient's interests, what services and activities are available locally that fit those interests and supports access to activities. Community Connector will accompany patients to a service or activity if required.  Up to 6 sessions  Holistic (3+) | Not specified | Global Assessment of Health - EQ-VAS Health Related Quality of Life - EQ-5D A&E and GP usage Mental Wellbeing - Short Warwick Edinburgh Mental Wellbeing Scale (SWEMWBS) |
| Dayson and Leather 2020 [62]  England  Grey Literature | Evaluation of Hale Community Connectors Social Prescribing Service 2018-2019 | Mixed methods evaluation | Stroke Diabetes Hypertension Raised cholesterol Asthma Depression Anxiety Chronic pain Arthritis Fibromyalgia   67% at least 1 LTC | Female 69% Male 32%  Under 25 5% 25-44 28% 45-65 37% 65-84 21% 85+ 8%  White British 51% Asian or Asian-British 26% Black or Black-British 1% Mixed 1% Unknown 21% | Anxiety and low mood Social Isolation Presence of LTC Money and debt support Frequent GP attender | Referral from a GP/practice staff/healthcare professional, followed by a home visit or meeting in a mutually agreed setting with a Community Connector who will work alongside the individual to establish what support is required and what they are interested in. The Community Connector helps identify what services and activities are available locally that fit those interests and support the individual to access them.   Up to 6 x 1-hour sessions  Holistic (3+) | Stroke Support Groups Speech and Language Therapy Equality together - home aids Community Occupational Therapy Assessment Art therapy for people post-stroke Accessible transport Advocacy and support for asylum seekers Theatre group Mixed ability sports Yoga Boxing Church activities Walking group Benefits advice Craft group at community centre Swimming Home support - cleaning, assistance to attend appointments Luncheon club Seated exercise Fibromyalgia support group | Health Related Quality of Life - EQ-5D HRQL Global Assessment of Health - EQ-VAS |
| Dayson et al. 2016 [55]  England  Grey Literature | The Rotherham Social Prescribing Service for People with Long-Term Health Conditions | Mixed-methods evaluation | Not specified  100% | Female 62% Male 38%  Under 50 6% 50-59 8% 60-69 14% 70-79 30% 80-89 35% 90+ 9%  White British 93% Asian 3.3% Black 0.2% White other 0.7% Unknown 3.3% | Presence of LTC | GP practices refer eligible patients and carers to the Voluntary and Community Sector Advisors, who undertake an assessment of support needs, typically during a home visit, before referring them on to appropriate Voluntary and Community Support services.  Not specified  Link Worker (3) | Information and Advice Community based leisure and social activities Befriending  Community transport  Complimentary therapy  Carer Intervention  Community activities coordinator Home based community exercise Advocacy and support  Counselling | Demand for hospital-based health interventions - Hospital Episode Statistics (HES) Wellbeing Star |
| Elston et al. 2019 [40]  England  Journal Article | Does a social prescribing "holistic" link-worker for older people with complex, multimorbidity improve well-being and frailty and reduce health and social care use and costs? A 12-month before-and-after evaluation | 12-month before-and-after study | 2 or more LTCs  100% | Female 73.3% Male 26.7%  50-59 5.8% 60-69 11.6% 70-79 26.7% 80-89 44.2% 90+ 10.4%  Not specified | Age 50+ Presence 2+ LTCs May benefit from social intervention | Patients referred from primary, community and secondary care to link worker for initial strengths-based, guided conversation, often in patients’ home, to determine need and decide whether signposting, a short conversation or a more in-depth holistic conversation is required. LW uses a range of tools over several meetings to enable the person referred to understand what matters to them and set goals for living well.   30-40 minutes sessions, up to 12 weeks  Link Worker (3) Holistic (3+) Secondary and Community | Resilience-focused coaching Practical support and advocacy to navigate and access local health, social and economic services | Wellbeing Star Patient Activation Measure (PAM) Warwick-Edinburgh Mental Health and Wellbeing Scale (WEMWBS) Rockwood Clinical Frailty Scale (RCFS) Contact with A&E, in-patient, outpatient, community, and social care |
| Esmene et al. 2020 [42]  England  Journal Article | Beyond adherence to social prescribing: how places, social acquaintances and stories help walking group members to thrive. | Case study | Diabetes (type 2)  100% | Female 50% Male 50%  18-26 1 (4.2%) 26-35 1 (4.2%) 36-45 1 (4.2%) 46-55 1 (4.2%) 56-65 6 (25%) 66-75 9 (37.5%) 75+ 5 (20.8%)  Not specified | Diabetes (type 2) | GP referred patients to walking group  N/A  Direct referral (2) | Walking group | N/A |
| Ferguson and Hogarth 2018 [53]  England  Grey Literature | Social Prescribing in Tower Hamlets: Evaluation of Borough-wide Roll-out | Mixed methods evaluation | Depression Anxiety Dementia Other mental health concern Cardiovascular disease Stroke Hypertension  Diabetes  Chronic Obstructive Pulmonary Disease Asthma Respiratory disease  Epilepsy  Chronic pain Arthritis  Cancer  Learning disability  Hypothyroidism  Kidney Disease   52% | Female 60% Male 40%  30-64 70% 65+ 12%  Bangladeshi/Bangladeshi British 48% White 23%  Black/Black British 12%  Mixed ethnicity 7%  Asian/Asian British: all other 5%  Other ethnicities 4% | Registered with participating GP  Expressed non-clinical support needs e.g. exercise, weight management, anxiety/stress/depression/low mood, social isolation, learning/training/employment, money/debt/benefits, housing issues, smoking, drugs, alcohol and other  addictive behaviours | Direct referral - referral from primary care indicates which service client requires and social prescriber made the onward referral with no direct contact with client. Social Prescribing referrals - Referral from primary care (inc. GPs, nurse, reception/admin, healthcare assistant, assistant practitioners, practice manager, psychologist/psychiatrist, pharmacist), or self-referral, to Social Prescriber, who conducts consultation via telephone or face-to-face depending on need, to identify support package. Referrals and signposts made to voluntary or community sector organisations or informal support groups, some referrals to statutory organisations. Motivational interviewing, goal-setting and coaching skills, and some SPs accompany clients to services. Feedback provided to referrers via EMIS, email, letter, or verbal at clinical meetings.  No limit to contact, most between 2 and 5 contacts of 30-60 minutes  Direct Referral (2) Holistic (3+) Self-referral to Link Worker | Health Trainers Social advice on housing, debt management and finances; IT support and training; management of energy bills; local skills exchange; employment/ volunteer advice; health, wellbeing and lifestyle support Exercise, weight management and healthy eating for patients with BMI >30 Community centre providing support for learning and skills development, help with employment and social welfare, legal advice and finances  Community centre offering predominantly services for women and older people Structured lifestyle programme for people with long term conditions Primary care psychology services  Educational community centres; libraries  Social services  Mental health, recovery and wellbeing services  Working with over 50s to improve social networks and activity Welfare, finance, housing and legal advice  Legal advice services Support with homelessness and housing  Energy saving support for vulnerable tenants  Community health service  Charity advice and befriending services for elderly patients Better Leisure Centres  Physical activity and socialising for over 50s | Measure Yourself Concerns about Wellbeing (MYCaW) |
| Giebel et al. 2021 [51]  England  Journal Article | A socially prescribed community service for people living with dementia and family carers and its long-term effects on well-being | Quantitative evaluation | Dementia  56% | Male 63.4% Female 35.7%  Ave. 74 +/- 8 years Range 57-90 Not specified | Presence of dementia diagnosis | Participants referred by GPs and psychiatrists at memory clinic, some self-referred, to the activity. Dementia care navigators (volunteers) also support the wellbeing of participants and signpost to wellbeing services. | Happy and Healthy classes based at local community and leisure centre, including low-impact exercises, local walks, Tai Chi, relaxation techniques, mindfulness, and games Quiet hours in the gym and swimming pool Low impact exercise Water-based exercise | Short Warwick-Edinburgh Mental Wellbeing Scale (SWEMWBS) |
| Howarth et al. 2021 [52]  England  Journal Article | Creating a transformative space for change: A qualitative evaluation of the RHS Wellbeing Programme for people with long term conditions | Realist evaluation | Not specified  100% | Not specified  Not specified  Not specified | Presence of 2+ LTCs Requires support for anxiety, improve confidence, to re-engage with others, to improve mental and/or physical health. | Most referred from Enhanced Care Team (primary care), small number from local social prescribing community connectors. Support from Wellbeing volunteers and a Therapeutic Horticulturalist.   N/A  Direct referral (2) Link Worker (3) | RHS Garden Bridgewater to help create a wellbeing garden - therapeutic horticulture | N/A |
| Joseph and Seguin 2023 [37]  USA  Journal Article | Something Fun to Look Forward to: Lessons From Implementing the Prescription for Health Farmers Market Initiative in Rural Upper Michigan | Mixed methods pilot study | Hypertension Diabetes (Type 2) Hyperlipidemia Obesity | Female 90% Male 10%  20-29 19% 30-39 14% 40-49 19% 50-59 14% 60+ 33%  Not specified | Self-reported desire to increase fruit and vegetable intake in diet | Referral to the program from a primary care provider to community health worker who delivered pre-program orientation, 'Prescription for Health' program card and health goal sheet, and distributed vouchers weekly.  Weekly  Link Worker (3) | Nature-based social prescription - Fruit and vegetable vouchers for local farmer's market Nutrition handouts Individual guidance on health goal setting | PROMIS Global Health Short Form v1.2  Biometric data inc. weight, BMI, and resting blood pressure Fruit and vegetable intake questionnaire Food literacy questionnaire |
| Kellezi et al. 2019 [54]  England  Journal Article | The social cure of social prescribing: a mixed-methods study on the benefits of social connectedness on quality and effectiveness of care provision | Mixed-methods study | Not specified  100% | Female 63% Male 32% Prefer not to say 1%  29-85 years Mean 60.4 years  White and/or British 84% | Presence of LTC  Experiencing loneliness | Referred by GP, practice nurse or self, to initial meeting for needs assessment with health coach, who prescribes self-care management or refers to link workers, who connect patients with relevant third-sector groups.   Weekly, up to 8 weeks  Link Worker (3) Self-referral to Link Worker | Not specified | No. of group memberships Community belonging - single item from population survey of social attitudes Loneliness - UCLA Loneliness Scale (ULS-8) No. of primary care visits |
| Kiely et al. 2021 [91]  Ireland  Journal Article | Primary care-based link workers providing social prescribing to improve health and social care outcomes for people with multimorbidity in socially deprived areas (the LinkMM trial): Pilot study for a pragmatic randomised controlled trial | Mixed methods pilot study | Kidney disease High blood pressure Diabetes Musculoskeletal problems Depression or other mental health problem  100% | Female 70% Male 30%  Mean 63yo  Not specified | Polypharmacy as proxy for multimorbidity, >=8 regular medications indicate 2+ LTCs | Eligible patients selected at random, checked by GP and referred to Link Worker for Initial meeting to identify needs, and refer to community resources. Intensity of support tailored to individual needs of patient, from support telephone calls to accompanying patients to activities.   Not specified  Link Worker (3) Holistic (3+) | Activities based within local community resources | Health related quality of life - EQ-5D-5L Hospital Anxiety and Depression Scale (HADS) Activities of daily living - Frenchay Activity Index Patient Activation Measure (PAM) Multimorbidity Burden of Treatment Questionnaire Investigating Choice Experiments for the preference of older people CAPability measure for Adults – ICECAP A |
| Kiely et al. 2024 [33]  Ireland  Journal Article | An exploratory randomised trial investigating feasibility, potential impact and cost effectiveness of link workers for people living with multimorbidity attending general practices in deprived urban communities | Feasibility randomised control trial | 2 or more LTCs  100% | Female 63%  Male 37%  Under 65 59%  Not specified | 2+ LTCs  5+ prescribed medications  Attending a GP serving a deprived urban community  Known social problems, addiction issues, mild mental health conditions, frequent attenders or those who did not attend health appointments.  18+ | GPs referred to a practice-based link worker, who carried out an initial assessment to identify participant’s needs and then connected participant to community resources and access support, with follow up contact.  1 month period  Holistic (3+) | Community resources | Health related quality of life (EQ-5D-5L)  Mental health -Hospital Anxiety and Depression Scale (HADS)  Wellbeing ICE-CAP A (ICEpop CAPability measure for Adults)  Cost effectiveness - Quality Adjusted Life Years (QALYs) |
| Kiely et al. 2024 [34]  Ireland  Journal Article | Implementing a General Practice-Based Link Worker Intervention for People with Multimorbidity During the Covid-19 Pandemic- a Mixed Methods Process Evaluation of the LinkMM RCT | Mixed-methods Process Evaluation | 2 or more LTCs  100% | Quant sample: Female 65%  Qual sample:  Female 69%  Quant sample:  65+ 43%  Qual sample:  65+ 31%  Not specified | 2+ LTCs  5+ prescribed medications  18+  Attending a GP serving a deprived urban community  Psychosocial issues  Deemed suitable by GP | GPs referred to a practice-based link worker, who carried out an initial assessment to identify participant’s needs and then connected participant to community resources and access support, with follow up contact.  1 month period  Holistic (3+) | Community resources for:  Health self-management  Mental health  Social activities and hobbies,  Health and fitness  Financial and legal support  Adults’ education and training  Housing and Homeless services  Addiction, alcohol and smoking  Older adults | Quantitative data on implementation and community resource referrals  Qualitative interviews |
| Loftus et al. 2017 [35]  Northern Ireland  Journal Article | Impact of social prescribing on general practice workload and polypharmacy | Quality-improvement study | Not specified  100% | Female 70.6% Male 29.4%  Mean 72.9 years (SD 7.3)  Not specified | Age 65 years+ Presence of LTC (including falls, social isolation, depression, and anxiety) Polypharmacy (5+ repeat prescriptions) Frequent GP attender. | Initial contact from GP to explain service to patient, who is then referred to Social Prescribing Co-ordinator, who contacted patient via telephone and home visit to select suitable activity programme.  Not specified  Link Worker (3) | Social clubs Men's Shed, Counselling Arts programme Falls prevention Exercises classes Crochet classes Personal development Craft classes Befriending Computer course | No of contacts with surgery, GP home visits No. of new prescriptions and no. of repeat prescriptions |
| Mercer et al. 2017 [36]  Scotland  Grey Literature | Evaluation of the Glasgow "Deep End" Links Worker Programme | Cluster randomised control trial with parallel Mixed methods process evaluation | High Blood Pressure Stroke/mini-stroke Diabetes Angina/Heart Attack Heart Failure Anxiety/Depression Arthritis Back Problems Thyroid Problem Eczema/Psoriasis Liver Disease Kidney Disease Asthma Chronic Bronchitis Migraine Cancer Irritable Bowel Syndrome  100% | Female 60% Male 40%  Mean 46 yo  Not specified | Mental health, physical health or social problems identified by GP | GP practice signpost to community organisations or refer patients to Community Links Practitioner who work one-to-one with patients. Practice-organised activities also available for patients.  Varied  Signposting (1) Holistic (3+) | Link to community organisations or practice-based activities such as walking groups or patient engagement groups | Health-related Quality of Life - EQ-5D-5L Investigating Choice Experiments for the preference of older people CAPability measure for Adults - ICECAP-A   Hospital Anxiety and Depression Scale (HADS) Work and social functioning - Work and Social Adjustment Scale Lifestyle behaviours - smoking, alcohol, exercise Healthcare utilisation - medication, hospital admissions, and GP practice contact |
| Mistry et al. 2017 [39]  England  Grey Literature | Prescription Plus Crawley: The Case for Project Expansion | Mixed methods evaluation of a short-term pilot study | Stroke Chronic Arterial Disease Chronic obstructive pulmonary disease Asthma  Chronic Heart Failure Arterial Fibrillation Mental Health Fall Risk Dementia Hypertension Depression Cancer Diabetes Kidney disease  100% | Female 70% Male 30%  18+  Not specified | Presence of LTC Frequent GP attender Additional non-medical needs | GP refers patients to Community Support Coordinator, who will see patients either in the GP surgeries, or at the patient's home for an assessment visit. Social prescription is coproduced with the Coordinator, using a menu of 37 local statutory, voluntary and community sector local organisations. Coordinator will accompany patient to the first session and follow up to encourage engagement. Patients can also be signposted to organisations not on the menu.   Not specified  Link Worker (3) Holistic (3+) | Social and leisure activities Information, advice, and practical support Mental health support and counselling Specialist support around conditions/circumstances Physical health and wellbeing Support with volunteering Social care | Healthcare usage - Risk of admission score, Hospital Episode Statistics, Secondary Care Activity Wellbeing Distance Travelled questionnaire |
| Moffatt et al. 2017 [92]  England  Journal Article | Link Worker social prescribing to improve health and well-being for people with long-term conditions: qualitative study of service user perceptions | Qualitative study | Diabetes (types 1 and 2) Chronic obstructive pulmonary disease Asthma Coronary heart disease Heart failure Epilepsy Osteoporosis  100% | 47% Female 53% Male  40-44 7% 45-49 10% 50-54 7% 55-59 17% 60-64 13% 65-69 17% 70-74 30%  White British 80% Black and minority ethnic 17% White Irish 3% | Age 40-74 years Presence of LTC | Referral from a primary care practitioner (GP, practice nurse, healthcare assistant) to Link Worker where meaningful health and wellness goals are jointly identified and service users are connected, when desired, to community and voluntary groups and resources.  Varied as required, for 2+ years  Holistic (3+) | Long-term condition management (voluntary sector  support groups) Mental health (CBT)  Physical activity (gym, walking group, swimming) Weight management/healthy eating  NHS services (physiotherapy)  Welfare rights advice (benefits advice, aids and  adaptations) Learning/employment assistance (CV writing)  Voluntary work  Arts-based activities (choir, art therapy)  Community-based activities (gardening, fishing, crafts) | Wellbeing Star |
| Moffatt et al. 2023 [31]  England  Journal Article | Impact of a social prescribing intervention in North East England on adults with type 2 diabetes: the SPRING_NE multimethod study | Multi-methods evaluation | Diabetes (type 2) Diabetes (type 1) Chronic obstructive pulmonary disease Asthma Coronary heart disease Heart failure Epilepsy Osteoporosis Anxiety  Depression  100% | Female 43% Male 57%  40-74 yo Mean age 58.16  Non-white 19% White 81% | Presence of LTC Age 40-70 years old | Primary care professionals refer to link worker for initial one-to-one contact and identify concerns, then co-production of a personalised action plan to address problems. Link workers supported clients to access a range of local services (e.g. physical activity classes and welfare rights) or to develop self-directed goals. Subsequent contact was either face to face or by telephone, text, e-mail, or video call.   As necessary, minimum every 6 months for 3.5 years  Holistic (3+) | Support in seeking employment Advice on benefits.  LTC management sessions Small community groups, such as information technology (IT), photography, cookery, gardening, exercise  classes and walking groups Social groups VCSE and NHS-run mental health support; local council fitness programmes volunteering opportunities | Glycated haemoglobin level (HbA) BMI Blood pressure Cholesterol level Smoking status Health-care costs and utilisation (QALYS) Health-related quality of life - EQ-5D-5L) Wellbeing Star |
| Munford et al. 2020 [45]  England  Journal Article | Effects of participating in community assets on quality of life and costs of care: longitudinal cohort study of older people in England | Longitudinal cohort survey | Asthma Cancer Back pain/Sciatica  Bronchitis Chronic obstructive pulmonary disease Kidney disease Colon/Irritable bowel Congestive heart failure Diabetes Hard of hearing Heart disease/angina High blood pressure High cholesterol Osteoarthritis Osteoporosis Overweight Poor circulation in legs Rheumatoid arthritis Rheumatic disease Stomach problem/ulcer/etc Stroke Thyroid disorder Problems with vision  100% | Female 52% Male 48%  65-69 years 32% 70-74 years 28% 75-79 years 21% 80-84 years 12% 85+ years 7%  White 94% | Presence of LTC Age 65+ | Part of a wider integrated care programme for older people that included a programme to improve use of community assets.   Not specified  Not specified | Group for elderly or older people (e.g. lunch club) Education, arts, music or singing group (including evening classes) Religious group or church organisation Charity, voluntary or community group Social club (including WMCs, Rotary Clubs, etc.) Sports club, gym, exercise, or dance group | Health-related Quality of Life - EQ-5D-5L Quality-adjusted life years (QALYs) Healthcare utilisation - no. of GP and hospital contacts Social value QALYS  Community asset participation |
| Palmer et al. 2017 [58]  England  Grey Literature | Social Prescribing in Bexley: Pilot Evaluation Report | Mixed methods evaluation of pilot study | Dementia Diabetes Chronic obstructive pulmonary disease Hypertension  100% | Female 52% Male 48%  25-34 1% 35-44 1% 45-54 4% 55-64 6% 65-74 19% 75+ 69%  Not specified | Struggling with significant life change  Struggling with their health conditions  Socially isolated  Frequent attenders of primary care/A&E  Presence of dementia  Carers  Carers who have long term conditions | GPs, healthcare practitioners referred to social prescriber who provides a mix of formal referring and informal signposting according to individual need. Clients can also self-refer. Social Prescriber meets clients at a mutually beneficial location, which includes home visits, GP surgeries, and community locations and supports access to a range of voluntary and community services and activities   Not specified  Link worker (3) Holistic (3+) Self-referral to Link Worker | Age UK - Men's Sheds, befriending, community support services, day centre, home support, memory cafe, pop in parlours, trusted tradesmen, volunteering Evergreen - befriending, home support care, trusted tradesmen, volunteering, clean team, knitters Carers Support - care navigation, befriending, carers wellbeing hub, day centre, emotional support, financial advice, respite, support group Mind - Nexus, health trainers, IAPT, mindfulness, peer support, recovery college, art group Alzheimer's Society - weekly activity group, dementia society, singing for the brain, carer info programme, dementia support Crossroads Care - befriending, memory cafe, monthly tea rooms, respite Bexley Libraries - activities, games club, IT buddies, reading group Irish Community Services - financial advice, lunch club Bexley Accessible Transport Scheme (BATS) Steps for Health The Learning Centre BVSC-Volunteering Cruse Bereavement Care University of the Third Age Bexley Spirituality Liver Disease Support Pulmonary Rehab Greenwich-Home Support Cruse Bereavement Care | Mental Wellbeing - Warwick and Edinburgh Mental Wellbeing Scale (WEMWBS)  Healthcare usage – primary, secondary and hospital contact |
| Panagioti et al. 2018 [46]  England  Journal Article | Is telephone health coaching a useful population health strategy for supporting older people with multimorbidity? An evaluation of reach, effectiveness and cost- effectiveness using a "trial within a cohort" | Trial within a Cohort study | Multimorbidity - 2+LTCs  100% | Female 54.4% Male 45.1%  65-69 years 25.3% 70-79 years 47.1% 80-98 years 22.6%  White 97.6% Non-white 1.8% | Presence of 2+ LTCs Needing some assistance with self-management | Embedded in a wider integrated care programme to improve care for older people with long-term conditions. Patients were recruited from within this cohort study, and referred to a health advisor for health coaching, social prescribing, and low-intensity support for low mood. Links to resources in the wider community through the community and voluntary sector via online self-assessment tool and resources, with links to relevant community resources and local support.  20 min monthly phone calls for 6 months  Holistic (3+) | Not specified | Self-management - Patient Activation Measure - short version (PAM) Quality of life -The World Health Organization Quality of Life brief measure (WHOQOL-BREF) Depression - The Mental Health Inventory (MHI-5)  Self-care- The Summary of Diabetes Self-Care Activities (SDSCA) Economic evaluation - EuroQOL 5-Dimension 5-Level (EQ-5D-5L) Economic Analysis - Quality-adjusted life years (QALYS) Healthcare utilisation - GP and hospital contacts |
| Pollard et al. 2023 [47]  England  Journal Article | Implementation and impact of a social prescribing intervention: an ethnographic exploration | Ethnographic exploration | Diabetes (type 2) Arthritis  100% | Not specified  Not specified  Not specified | Middle to early older age  Presence of at least one of 6 qualifying LTCs | Referral from GP practice to a link worker, who helps clients assess their current situation, including lifestyle and money, to agree a personalised action plan, following which the link worker supports patients to access relevant local community services, or in some cases, to support them to develop self-directed programmes.  Up to 4 years   Link Worker (3) Holistic (3+) | Benefits advice Gym - circuits class VCSE Diet-related services | N/A |
| Polley et al. 2021 [64]  England  Grey Literature | Tandridge District Council Wellbeing Prescription Service Evaluation Final Report | Mixed methods evaluation | High blood pressure Diabetes (type 2) Depression Arthritis Pre-Diabetes Anxiety High Cholesterol Asthma Coronary heart disease COPD Stroke Neurological Condition Dementia Cancer Learning disability Diabetes (type 1)  100% | Female 63% Male 37%  19-24 2.8% 25-34 7.6% 35-44 11.7% 45-54 16.8% 55-64 20.7% 65-74 19.0% 75-84 14.0% 85+ 6.9%  White 86% Asian or Afro-Caribbean 12% Not stated 2% | Require support with: Lifestyle and modifiable risk factors (weight management, getting more active, reduce alcohol consumption, stop smoking, substance misuse support) Emotional/mental wellbeing Social contact Independent living Carer support Finance and housing Covid-specific reasons | Referrals from GP, practice nurse, self-referrals, community health and care, adult social care, local community providers to Wellbeing Advisor for face-to-face appointments or home visits.  Not specified  Holistic (3+) Self-referral to Link Worker Community | Modifiable risk factors - weight management, getting more active, reduce alcohol consumption, stop smoking, substance misuse support Social Contact Emotional/mental wellbeing Clinical and illness support Independent Living Learning and Development Carer Support Finance Housing Covid-19 specific | Weight  BMI  Physical Activity - General Practice Physical Activity Questionnaire (GPPAQ) Mental Wellbeing - WEMWBS Alcohol Risk - Alcohol Use Disorders Identification Test (AUDIT-C) Measure Yourself Concerns and Wellbeing (MYCaW) Health Service Usage - visits to GPs, A&E and hospital |
| Simpson et al. 2020 [49]  England  Journal Article | Supporting access to activities to enhance well-being and reduce social isolation in people living with motor neurone disease | Qualitative analysis of pilot study | Motor Neurone Disease  100% | Female 60% Male 40%  40-79yo Mean 56 yo  Not specified | Recent retirement Need to get out of house and meet new people Opportunity to engage in activities Find purpose and inclusion | Patients were recruited by MND association visitors, public advisors, outpatient therapists and council-based occupational therapists. Patients are supported by an occupational therapist and link worker to identify and access community-based activities.   Varied  Link Worker (3) Secondary and Community | Not specified | Interviews |
| Wakefield et al. 2020 [48]  England  Journal Article | Social Prescribing as "Social Cure": A longitudinal study of the health benefits of social connectedness within a Social Prescribing pathway | Multi-method longitudinal study. | Not specified  100% | Male 45% Female 54% Unknown 1%  Mean 52.74 SD 14.79  Not specified | Presence of 1+ LT physical or mental health condition Feeling isolated, lonely, or socially anxious | Referral made by GP/practice nurse/self-referral to a Health Coach, who either recommends self-care management, or refers the patient to a community-based Link Worker (LW) who connects patients to relevant voluntary/community groups.  Regularly for progress monitoring  Direct referral (2) Holistic (3+) Self-referral to Link Worker | Local voluntary and community groups | Number of group memberships Community belonging - Hayward 1-item Social support - Haslam 4-item  Loneliness - ULS-8 Health-related quality of life - EQ-5D |
| Wildman and Wildman 2021 [44]  England  Journal Article | Evaluation of a Community Health Worker Social Prescribing Program Among UK Patients with Type 2 Diabetes | Cohort Study | Diabetes (types 1 and 2) Chronic obstructive pulmonary disease Asthma Heart failure Coronary heart disease Epilepsy Osteoporosis  100% | Female 43% Male 57%  Mean 57.7 years  White 82% Non-white 18% | Age (40-74) 1+ LTC | Primary care staff refer patients to community health worker for face-to-face appointment in a community setting, for individual assessment to identify condition management, motivational interviewing and action planning, identification of social needs goals and provides support and guidance to access to voluntary and community sector to address goals.   Varied (frequent and intense to occasional and brief, depending on need), up to 2 years (longer with link worker discretion)  Holistic (3+) | Welfare rights Employment support Housing advice Health and lifestyle support | HbA1c level |
| Wildman et al. 2019 [50]  England  Journal Article | Service-users' perspectives of link worker social prescribing: a qualitative follow-up study | Qualitative follow-up study | Diabetes (types 1 and 2) Chronic obstructive pulmonary disease Asthma Coronary heart disease Heart failure Epilepsy Osteoporosis  100% | Female 46% Male 54%  40-44 10% 45-49 10% 50-54 7% 55-59 17% 60-66 10% 65-69 20% 70-74 27%  Not specified | Not specified | Primary care practitioner refers patient to link worker (initial contact via telephone, appointment face-to-face) who help patients identify which areas of their lives they wish to change and how. May accompany patients to activities  Varied, as required, up to 2 years  Holistic (3+) | Weight-management groups Welfare rights Advice Arts-based activities) Return to work Volunteering opportunities | Wellbeing Star |
| Organising Groups:  Kensington & Chelsea Social Council and NHS West London Clinical Commissioning Group 2018 [66]  England  Grey Literature | Self-Care Social Prescribing | Mixed methods evaluation | Cancer Dementia  100% | Not specified  65 years+  Not specified | Age 65+ Presence of at least 1 LTC | Patients identified in primary care. Patient allocated to practice-based Health and Social Care Assistant (HSCA) or Case Manager (CM), who conducts assessment including recording outcome measures, goals, and makes direct referrals to directory of services.  Pre and post activity/service  Holistic (3+) | Physical & Exercise activities -African Dance -Escorting -Exercise at Home -Massage Therapy -Supported Gym Sessions -Walking support Mental wellbeing and reduced isolation  (non-dementia specific) -Befriending -"Link Up" activities  -Memory Cafe -Men’s Only Club Activities  -Carers Support Group Dementia-specific support -Arts and Culture in The Community (for dementia) -Creative and Cultural befriending -Dementia One-To-One Support N.B. Exercise at Home and Walking Support are also  provided to dementia referrals with a tailored approach Safety & Welfare Information and Advice -De-cluttering at home (can also be considered as part of mental wellbeing services) -Safety and falls prevention at home -Information and Welfare support advice Health Education & Nutrition -Healthy Lungs -Macular Degeneration Group -Nutrition and community lunches | Social Return on Investment (SROI) Health related Quality of Life - EQ-5d Mental Wellbeing - Warwick-Edinburgh Mental Wellbeing Scale (WEMWBS) Depression - Patient Health Questionnaire (PHQ-9)  ICEpop CAPability measure for adults (ICECAP-A) General Measure of Health - Short Form 12 (SF-12) Patient Activation Measure - PAM Quality Adjusted Life Year (QALYS) Social Care Outcomes - Adult Social Care Outcomes Toolkit (ASCOT) |
| Organising Groups:  Family Action, Healthy London Partnership and Apteligen 2018 [59]  England  Grey Literature | Social Prescribing in Secondary Care Pilot Service Evaluation Report | Mixed methods evaluation | Diabetes High blood pressure High cholesterol  Not specified | Male 47% Female 49% Unrecorded 4%  Up to 20 5% 21-30 3% 31-40 5% 41-50 4% 51-60 9% 61-70 8% 71-80 18% Over 80 44% Unknown 4%  White British 26% Black/Black British - Caribbean 6% Black/Black British - African 4% Black/Black British - Other 4% White - Turkish 3% White - English 2% White - European 2% White - Other 2% Asian/Asian British - Indian 2% Asian/Asian British - Bangladeshi 1% Asian/Asian British - Pakistani 1% Asian/Asian British - Other 1% Black African 1% White - Turkish Cypriot 1% | Social problems Isolation Information/advice Housing Financial problems Mild-moderate mental health problems Adults' relationship Destabilising Event Neighbour/community issues Risk homelessness Mental health of parent Education issues No-clinical activities | Referral made from health practitioners, GP, partner charities or self-referral during or after discharge from secondary care or hospital to Link Worker for holistic assessment to coproduce health and wellbeing plan and referral to community services, support agencies or statutory services. Support provided by volunteer befrienders if required.  Between 2 and 8 one-to-one sessions for 6-month period of pilot project  Holistic (3+) Secondary  Self-referral to Link Worker | Housing Issues Social Isolation & befriending Service  Benefit Issues  Activity & Exercise  Anxiety & Emotional Support Services Advocacy - Education & Learning  Other organisations/ agencies - Hackney People First, Connect Hackney, Triangle Stroke Project, Dogs Trust, Derman | Wellbeing Star HACT Wellbeing Value Calculator |
| Organising Groups:  MacMillan Cancer Support 2018 [61]  England  Grey Literature | Macmillan Social Prescribing Service Summary Evaluation Report | Mixed methods evaluation | Cancer  100% | Female 57% Male 43%  51-70 52%  White British 39% Black/Black British 27% Asian/Asian British 18% Other 15% | Cancer diagnosis | Referrals from outreach, primary and secondary care, community partners and self-referral to Link Worker for different levels of care:  Level 1 - telephone contact made with  patient to introduce the service and assess needs, signposting and referral is offered as needed, and/or the client is booked for a face-to-face level 2 session Level 2 - face-to-face hour long assessment in community setting where the client wellbeing is explored, and goals are identified. Actions are then agreed, and referrals or signposting is offered  Level 3 - clients may have further face-to-face sessions of an hour each, providing any additional support required.  Up to three, or five for complex cases, one-hour face-to-face sessions  Link Worker (3) Holistic (3+) Self-referral to Link Worker  Secondary care | Advice services, including financial support and food referrals  Physical activity  Treatment and health options, including addressing physical concerns and complementary therapies Support groups  Emotional wellbeing  Socialising and activity groups  Yoga  Learning, volunteering and return to work  Case work and information  Practical support  Cancer centre (unspecified)  Other | Measure Yourself Concerns and Wellbeing  (MYCAW) for Level 2 and 3 clients only Wellbeing - ONS Wellbeing Scale Social Return on Investment (SROI) |
